# Henry A. Smith, A.M., D.D.S.

**Published:** 1913-10-15

**Authors:** 


### OBITUARY.

### HENRY A. SMITH, A.M., D.D.S.

Dr. H. A. Smith, dean of the Ohio College of Dental Surgery, died at his home in Cincinnati, September 11th, 1913. Dr. Smith has been in poor health for the past two years and his death is not therefore unexpected. He was 81 years of age. He has had a long and prominent professional career. He began the study of dentistry with Dr. George Keeley of Oxford, O. and graduated from the Ohio College of Dental Surgery, March 6th, 1857. He associated himself with Dr. Charles Bonsall, a prominent dentist of his time, and eventually succeeded Dr. Bonsall in his practice. When he began the practice of dentistry in Cincinnati there were only ten or twelve practitioners in the city, and at this time there are nearly 300. During his life as a dentist he has seen the profession develop from an unorganized craft to a scientific body with high professional attainments and ideals. In its development he has had a large and important part. As a dentist he was thoroughly conscientious and highly skillful. He had a pleasing address and his patients were loyal to him. It used to be said of him by his confreres that his pa ients never went shopping. He was not a scientific student, but he was a close reader of his professional literature and had an excellent memory. He was a constant attendant of dental societies and always took prominent part in the discussions. He contributed many papers to the Old Mississippi Valley Dental Association, the Ohio State Society and the National Association, as well as to many other dental organizations. In 1878 he became dean of the Ohio College of Dental Surgery, succeeding Dr. J. Taft, who had accepted the deanship of the College of Dental Surgery of the University of Michigan. Dr. Smith had been chairman of the Board of Trustees of the College for several years and was familiar with the conditions of education at the time, although he had not taught in the College. He immediately undertook the task of increasing the attendance and effectiveness of the instruction. It was not long until larger quarters were required and the school was moved from the old building in College Street to the Central Avenue location from whence it was removed last year to its present home at 7th and Mound Street. The work Dr. Smith has done to maintain and place in the front rank of educational institution this school will never be known, except to a few who have been associated closely with him. His work for his own school naturally lead him to take prominent part in the deliberations of the National Association of Dental Faculties. Here he took a leading part always and was one of the strong personalities in moulding the career of that useful organization. He gave much of his time and energy to the cause of dental education throughout the country. As a teacher he has occupied the chair of Operative Dentistry with much credit and to the entire satisfaction of hundreds of students who have attended his classes during the past thirty five years.

Dr. Smith's leadership in educational affairs often brought him into spirited debates as to inter-colligate relations and he did not always look upon methods in the same way that others saw them, but he was always courteous and did not make enemies of his associates. He had the same experience with his nearer professional relations. Those who knew him intimately believed in him and stood by him in the most confidental relations. His faculty changed but little in all the years of his management, and he has always had the most amicable and loyal support of his associates, some of whom have continued with him since he assumed the deanship. He has done the profession a large service and he will long be remembered for the interest and generous contributions he has given to the dental profession. His personal friends throughout the country will learn of his death with sincere sorrow. He had a long life as he was in his 81st year, and 55 of these years were devoted to the practice and teaching of dentistry. What the world in general and the people with whom he came in active contact owe to such a man it is difficult to state, but men of such wide influence are not adequately appreciated or honored while living.

We are glad however always to take notice of the worth and good qualities of those whom we have known and honored in this life when they have passed on to the other life. In the case of our friend we cannot help wondering what more he could have done to have made his life more complete and commendable.

